# Knowledge

**DOI:** 10.1007/s12064-017-0242-5

**Published:** 2017-02-22

**Authors:** Jürgen Jost

**Affiliations:** 1 Max-Planck-Institut fur Mathematik in den Naturwissenschaften, Leipzig, Germany; 2 Santa Fe Institute for the Sciences of Complexity, Santa Fe, USA

**Keywords:** Knowledge, Information, Representation, Gestalt, Structural knowledge, Self-reference

## Abstract

We investigate the basic principles of structural knowledge. Structural knowledge underlies cognition, and it organizes, selects and assigns meaning to information. It is the result of evolutionary, cultural and developmental processes. Because of its own constraints, it needs to discover and exploit regularities and thereby achieve a complexity reduction.

## Introduction

The question of knowledge has been one of the guiding themes of the intellectual life of Olaf Breidbach. The systematic questions are

1. What is knowledge?
2. What can we know?
3. Where does knowledge come from?

He approaches these questions from many different, but interwoven perspectives:

- Epistemology/philosophy
- Theory of science
- Evolution
- Neurobiology
- Simulations in artificial neural networks
- History of science
- History of culture and art
- Media theory, analysis and conception of collections and exhibitions.

He devotes many publications and several monographs to this topic, such as Breidbach (2001, 2005, 2008), Breidbach and Vercellone (2011) and in particular Breidbach (2011), with his striking thesis that self-reassurance, becoming certain of one’s position, is only possible through the insight that one is the result of a contingent, autonomously evolving, historical process. In order to ground your knowledge, you need to realize not only that it depends on a contingent historical path, but also understand how such a process can create knowledge. This is the only way out of the inner perspective to which both a cultural tradition or the brain of a beetle are confined.

Of course, we should first clarify what we mean by knowledge. We are not concerned here with factual knowledge, with knowledge about the truth of propositions. That is a classical philosophical topic; a recent contribution to the old debate is, for instance, Williamson (2000) where many of the classical arguments are discussed and often refuted. The issue is whether knowledge can be defined as something like justified true belief, in which case the notion of belief would be prior to that of knowledge. But this is not our concern. We are not interested in direct knowledge about isolated facts; in fact, it is not even clear whether the concept of an isolated fact is even meaningful, and for instance, Quine (1953) has argued that the empirical content of an individual statement is not a meaningful concept. In this essay, we shall reach similar conclusions, from a different perspective and combining tools from the formal sciences with insights gained from neurobiology and cognitive psychology, and therefore perhaps better supported than a merely philosophical approach.

Instead of factual, declarative knowledge, we are rather interested in structural knowledge, that is, in schemes for the organization of clusters of data that can guide interactions with the outside world. For instance, when we ask what the beetle’s brain knows about the world, a first answer might be that it “knows” how to behave in specific situations. But even this answer cannot really be correct. Insects have evolved as couplings of very general effectors with rather specific actuators. This means that one and the same general control mechanism can trigger a wide range of specific actions, according to body segment, insect species, developmental stage, or caste in social insects (see for instance Breidbach and Kutsch 1990). Of course, this is very different from human procedural knowledge, or from the cultural traditions of a society. Nevertheless, we can ask whether there are any general principles underlying or governing these diverse manifestations of knowledge.

This essay is an expanded and adapted version of the English translation of a text that I had written many years ago to be published in a volume on knowledge that Olaf Breidbach had planned to edit. It is the result of an intellectual exchange spanning several decades. It draws upon such fields as the theory of information, statistics and learning theory, or the mathematical theory of dynamical systems, which are complementary to the fields mentioned above, but it reaches conclusions that are quite compatible with what Olaf Breidbach had found from his perspectives. It seems therefore most appropriate to dedicate this essay to his memory.

## Knowledge as representation

What can we know? This question is very naive, and we shall expose its naiveté. Nevertheless, it is instructive to turn to it in detail, starting in a negative manner by determining what we cannot know. There is a lot that we cannot know: we cannot predict the lottery numbers for next week, and often not even the weather. There exists a standard explanation: such phenomena are based on so-called chaotic dynamics, i.e., those in which small differences in the initial conditions grow exponentially over time. So according to this explanation, the problem is that we cannot determine the initial states with absolute precision. But why not? If we could go down to the molecular level, or the nuclear, perhaps even the subatomic one, we should be able to gain the required precision. However, then the required effort will become immense, and this can, in fact, be captured in a more principled and basic way.

But let us proceed with our example for a while. We can know the weather of the last week, because we may remember it or because someone has recorded it. But how is it with the weather in Leipzig, or more precisely in the swamps in the lowlands of Elster and Pleiße on 10/08/1016? (Where we suppress the fact that there has a calendar reform between now and then, and so, the correspondence between the dates is more complicated.) There exist no sufficiently precise documents, and the backward computation faces similar problems as the forecast of the future. But why then do physicists believe that they can reconstruct the first 3 min^1^ of the universe, that is, the situation immediately after the Big Bang more than 10 billion years ago? And if we already have great difficulty with the weather next week, why then are the predictions of climate scientists for global warming over the next decades at all credible?

In order to analyze the problem in a more fundamental manner, we look at a different type of example, the cellular automata introduced by John von Neumann and Stanislas Ulam, see Von Neumann and Burks (1967).^2^ In the simplest variant, one has a chain of elements which for each discrete point in time, $n=0,1,2,3,\ldots$, can assume one of two possible values, 0 or 1, off or on, black or white—the semantics is arbitrary. Which of these two values an element takes at time *n* is calculated according to a fixed rule from its own value and those of its neighbors at time $n-1$. One specifies some initial values at time 0 and then lets the dynamics run on its own as described. The dynamical rule is completely deterministic, and we can therefore ask whether from the knowledge of the initial values, we can know the state of the elements at a given later time, for example, $n=1000$. Of course, we can simply let the system run and then observe what happened after 1000 steps. But the question is whether this is really necessary, or whether we can compute the answer directly from the initial values and the deterministic iteration rule. For some rules, this is trivially possible, for example, when the rule simply always assigns the value 0. It is much more interesting, however, that there are rules for cellular automata, in which there is no shorter or easier way to obtain the result after 1000 steps, than to let the dynamics operate itself for 1000 steps. The complexity of the system is thus not reducible. In our weather example, this would mean that there is no easier way to determine the weather for the next week than to watch the weather for 1 week. We cannot know the future in advance. Laplace was just simply too naive.

The situation seems to be even worse: if in quantum chemistry or molecular biology, one wants to compute the three-dimensional shape of some molecule, such as a large protein, one often has to run a huge supercomputer for hours or even days, and sometimes still this does not even deliver the correct result. The underlying equations are so complicated or have so many degrees of freedom that such an enormous computational effort cannot be avoided. The computation of the system, in this case of a simple molecule, is many orders of magnitude more complex than the simple observation. Why is that? Among other things, despite all the impressive miniaturization achievements of semiconductor technology, the computer is still operating on a much coarser scale than the molecular or atomic one, which is that of the object in question. By now there are at least theoretical designs for quantum computers, operating at a scale that is even smaller than that of the molecule. And modern physics believes, according to superstring theory, to have identified the fundamental, no longer resolvable constituents of matter, which are many orders of magnitude smaller than the known elementary particles such as protons or electrons. If one were able to also compute at this most fundamental level, the computation would have overtaken the reality again. But then we would have still the problem that the computation would not be easier than the reality. Why would it be worth then to compute at all? The answer lies in a different direction. With the supercomputer, we do not compute or simulate a protein because we are interested in this specific protein as an individual object, but because all proteins with the same building blocks behave also similarly. There are physical laws and therefore regularities. So we only once have to compute and then know the result for all such proteins. That is the basis of our knowledge. Knowledge is based on the knowledge of laws and regularities, and thus it requires a regular world. This was known^3^ already to Leibniz and Kant, but it is reassuring that this insight has not been overturned by modern physics.

The Heisenberg uncertainty principle of quantum physics teaches us that an accurate prediction of all details of atomic processes is fundamentally impossible. But the Schrödinger equation incorporates a deterministic behavior of probability amplitudes. So while the classical behavioral of the quantum mechanical variables is not deterministic, but stochastic, conversely stochastic variables evolve according to deterministic rules.

But it gets even better: in most physical processes, or abstractly, in most dynamical systems there are only few relevant degrees of freedom, while all other degrees of freedom exponentially approach a state of equilibrium or average out, and then no longer influence the dynamical evolution. It can be shown mathematically that this behavior is typical of dynamical systems,^4^ see for instance Jost (2005). So we need to know even less than all the details of the initial conditions in order to predict the global behavior of the system. Where the cannonball strikes, depends only on few macroscopic variables, and we do not need to know the exact details of the internal molecular configuration or even the subatomic particles inside the ball. Apart from certain relativistic corrections, the trajectories of the planets around the sun follow the laws discovered by Kepler and the rules of Newtonian point mechanics. Thus, objects that are internally as complex structured as the planets can be treated as extensionless mass points for their celestial motion. Although the weather apparently behaves really chaotically, the underlying chaotic attractor has a particular dimension, which is substantially lower than the number of available degrees of freedom. While this does not allow for a concrete accurate prediction of all the details, it nevertheless constrains the possible dynamical evolution. In addition, on a larger time scale random fluctuations and chaotic perturbations average out, and this may perhaps allow for long-term climate predictions. This averaging of random fluctuations is incidentally also crucial for the transition from the stochastic behavior of the atomic quantum world to the deterministic dynamics of macroscopic processes. An even more significant confinement of the details of a finer scale takes place in the constitution of the atomic world from its elementary building blocks, and the latter can be isolated, if at all, only with enormous experimental effort in gigantic particle accelerators and colliders.

We have thus dealt with the physical and ontological aspects. In this sense, knowledge is possible because the world is regular and the dynamical behavior of macroscopic objects is typically determined by a few parameters.

We need to insert an important caveat here: such simple regularities as the laws of Kepler of planetary motion are the exception rather than the rule, as argued in Jost (2015b). At both smaller and larger scales, the physics gets much more complicated. And more generally, if the world were so simple that we could completely understand it, it would not be rich and complex enough to support our existence as physically quite complex beings.

Nevertheless, for our present purposes, the fact that at some scales, however rare and exceptional they may be, such simple regularities hold, is what makes predictions possible and therefore, as we are arguing here, can ground knowledge.

Thus, the present conclusion is that knowledge can represent the regularities of the outside world. But does the representation of a regularity not already presuppose a regular structure? Or, putting it another way, how is this knowledge then represented in turn? That is our topic.

## Information and knowledge

How do we gain knowledge? We interact with the outside world and with our fellow human beings, receive signals, inputs, messages. From this we need to build our knowledge. To understand this, we must first investigate what insights we can gain from a message and what internal conditions are needed for this. This leads us to the subject of this paper.

Shannon quantified the information of a message by its novelty for the receiver, how much it contributes to the reduction of the latter’s uncertainty Shannon and Weaver (1949). The information contained in a message is the greater, the more unexpected it is for the receiver. In this way it could be quantified which amount of information can be transmitted without interference by a given channel. A prerequisite of this approach is that a code between sender and receiver is arranged in which the message is written. In this approach, the meaning of the message does not need to be considered. In particular, in principle everything can be reduced theoretically to the simplest possible code, the binary one. A message is thus a sequence of 0s and 1s, and the receiver expects different such strings *s* with different probabilities *p*(*s*), and the average negative logarithm of these probabilities, that is, the entropy of the ensemble of possible messages, becomes the Shannon measure of the information,

1 $$\begin{aligned} H=-\sum _s p(s) \log _2 p(s) \end{aligned}$$

(where the base 2 logarithm is taken, so that when one of two equally probable alternatives, i.e., $$ p(i) = 1/2$$ for $$i=1,2$$, is observed, the information gain is 1 bit). This proved to be an extremely fruitful approach to understanding and optimizing the transmission of information, but the situation underlying Shannon’s theory is obviously not without assumptions. How does the receiver know the code of the sender? Had this code also been transmitted via the shared channel, and which other code had then be used for this transmission? This reduction obviously would not work. And how does the receiver know the probability distribution for the ensemble of possible messages? Probably from an empirical frequency distribution of already received messages. But this would move the question only into the past, instead of answering it.

Shannon’s theory is not concerned with the information as such, but with its transmission in a not further questioned context. Therefore, it focuses on the channel. However, our questions are aimed at the receiver and therefore cannot be answered within this theory. Their further analysis will lead us to the concept of knowledge. Because for these questions, the channel is not essential, the sender will also lose its specific role. The sender will only come back into view when it comes to strategies of influencing the receiver. But the latter, the receiver, has to be constituted, that is, it must be ensured that this agent will respond to the message at all, and this will in turn be expected only when he/she attaches a meaning to this message. So we ask what makes the information to an information for the receiver. And why does this information interest her/him? And is he/she interested at all in any information received? Is not some sequence of 0s and 1s, or some message written in some other code, not simply a random string without any discernible significance for the receiver, some noise that he/she no longer perceives, but rather ignores?

These questions suggest that we have to proceed differently for our purposes. If we want to understand the receiver, we cannot separate information and meaning. We have to start with the assumption that the system that we want to consider as the receiver of a message can perceive a certain difference. This means that its receptors or sensors can distinguish at least two different states of its external world. Such a perceived difference then becomes relevant for the system when those possible states have different consequences for the system, or, as Bateson (1972) put it succinctly, “a difference that makes a difference”. This can be expressed in a different response of the system, but it is sufficient if different internal states of the system arise. These states need not be visible to the outside world. A (perceived) difference causes a difference (in or for the system). Conversely, the difference is only perceived because it has a consequence for the system. Otherwise it would be ignored, disregarded. The specific reaction must have an advantage for the system over the indifference.

For this, while not unconditional, but simple situation, there are now two major extensions. The first takes us to the theory of signs (see for instance Keller 1995) and semiotics (e.g., Eco 2002). The perceived difference need not be directly relevant to the system, but can be a sign, that is, represent a different distinction. Following Peirce’s classification, the system can, instead of directly perceiving the raindrops, interpret a dark cloud as a symptom of the expected rain, a notched circular gray spot with parallel slashes in the weather forecast as an icon^5^ or the verbal communication “It will rain” as a linguistic symbol. It can then anticipate the rain by a causal, associative or rule-based inference according to the type of sign received.

The meaning of the sign consists then, as already stated, in its effect on the receiver. In particular, this distinction between the various types of signs and inference schemes does not matter. We can thus also bring the sender back into the picture if it just produces the signal for the purpose of influencing the receiver. The difference caused by or resulting from the perceived difference in, by or for the receiver then needs to make a difference for the sender. Extending Bateson’s slogan, we could speak of a difference that makes a difference that makes a difference.

This is no longer so easy, and in particular not without assumptions and preconditions. We turn therefore to the second extension of the basic situation described. The receiving system is no tabula rasa, not a primitive stimulus-response mechanism, but it possesses an internal structure and is shaped by the information already received in the past. However, this aspect must be further refined. First, a temporal factor comes into play. The received character may perhaps only point to a relevant difference in the future. The flowering shrub will bear fruit in a few weeks, and it is useful to remember it then. This requires a memory which can store information and make it currently available. Thus, the system must be able to bridge a time difference. This stored information is the simplest form of knowledge. Although the information has novelty value for the system, it does not lead directly to an external reaction, but first creates a different internal state of the system. The question of the storage medium will be postponed. In any case, this represents only the simplest form of memory. In the next stage, the memory can be used to compare a current stimulus, a signal, a message to another one already received in the past. Thus, the system builds its own internal reference system. What is new can then be set in relationship to what is already known. The system can thus also detect differences between differences, i.e., notice that a new stimulus differs from the previously received ones. So the system is gaining not only the information from the current stimulus, but also information about the statistical distribution of stimuli. So one of the issues raised seems to be easily solvable. It is important that now a stimulus contains information on various levels, first about what can be extracted from it via causal, associative or rule-based inference, secondly, on a longer time scale about an ensemble of stimuli. This can of course be iterated to gain more, higher levels. One aspect of this is that this current stimulus needs to be recognized as a member of an ensemble. Thus, it has to confirm expectations of the system at a higher level. At the same time, on a more elementary level it has to have some novelty value and therefore must carry information, and so should not be fully expected, but should have an element of surprise. Of course, this balance between expectation confirmation and surprise shifts in the course of system development. This occurs on one hand through the fact that the new stimulus may affect and modify the distribution of the stimulus ensemble, and on the other hand by the described development of higher levels of organization. Knowledge is not only produced, but also structured. We therefore consider knowledge as stored and structured and thus available information.

The information is received and “evaluated”, thus loses its value, which consisted precisely in its novelty. In contrast, the knowledge gains value by the potential contained in it for a future application and as a reference for new information that can only acquire their value by the very fact that the structure of knowledge gives them internal meaning.

## Generation of knowledge

Now two aspects have been mixed, which have to be separated again. This becomes clear if we undertake it to assess or evaluate that knowledge, as we did with the information by its novelty value.^6^ Knowledge was introduced as stored information about the outside world and in that capacity, it was also assessable as information. We thus measure to what extent uncertainty about the state of the outside world is reduced by that knowledge. This also leads to a problem that is, however, only indicated here. The quantification of information or knowledge by an entropy says nothing about the value of knowledge for the respective system.^7^ In any event, knowledge as a collection of data about the outside world constitutes a complexity gain for the system. This gain consists in the fact that for the system, the complexity of the outside world is reduced by enabling it to make certain aspects of the outside world reproducible as regular (see Jost 2004 for a systematic analysis).

It is more important for our purposes, however, that the production of knowledge is not a passive storage process, but an active structuring process. The efficiency of the patterning is then expressed at a given data collection by a complexity reduction. Conversely, such complexity reduction through increased efficiency of structuring data then in turn sets capacity free for an extension of the amount of data, that is, an increase in complexity. This interplay can be expressed only when we distinguish between external and internal complexity, as in Jost (2004).

We will now analyze this active structuring process in more detail, particularly in the context of human cognition. This is about discovering and exploiting regularities in the data obtained. Before we examine this more closely, however, it must be pointed out that this in no way represents a first-principles situation. What an (e.g., sensory) datum is, will only be determined by the internal structure of the system, in the context of which it can get assigned meaning, usually on the basis of specific observations selected by internal hypotheses (see Jost 2004).

This aspect of the feedback, that the internal structures and hypotheses determine what is singled out and perceived from the abundance of sensory stimuli and how this develops into percepts, is probably the most important one for the understanding of cognition. Nevertheless, we want to first investigate the supposedly simpler direction, namely the creation and adaptation of internal models from observations. After the failure of most rule-based models from AI, artificial intelligence, for orientation in situations that are not precisely circumscribed and detectable, the theoretical approach has shifted (among many references, see for instance Pfeifer and Scheier 1999; Ay et al. 2012 or the collection of essays in Engel et al. 2015).^8^ The difficulty that the AI research could not overcome was to formalize the respective required context or background information and to incorporate it into logical chains. Even in situations where this contextual knowledge simply consists of a fixed set of simple rules, high performance could be achieved only through the use of extremely high computing capacity, as in chess, without being able to reproduce the intuitive position sense of human players. In Go, the problem was that purely rule-based circuit chains apparently are not able to develop global long-term strategies within which local positions can be evaluated. Therefore, the computer programs remained hopelessly inferior to good human players, until recently a deep neural architecture that gained expertise by playing against itself could beat the world champion (Silver et al. 2016). Formal rule-based methods are completely overwhelmed in real environments, whose context is too complex in principle for being completely representable within the system. This becomes already clear from basic system theoretical considerations, see Jost (2004). More recent approaches therefore are no longer rule-based models expressed in terms of formal logic, but rather explore different types of statistical learning and inference or try to replace the deductive sequential reasoning by recurrent association dynamics. We lose the certainty that arises from the formal correctness of a chain of logical conclusions, but will gain the chance to get a usable and generalizable model. But this paradigm shift does not simply consist in replacing one class of strategies for solving a given problem by another, but the problem as such is also shifted fundamentally. Although we have already indicated and will come back to the fact that this does not yet yield a really adequate formulation of the problem of cognition, we shall briefly discuss those approaches because they provide some useful insights for understanding the origin of knowledge.

The starting point is that precisely what is assumed without question in the Shannon information theory, namely, the statistical distribution *p* of possible input signals, has to be reconstructed, or at least estimated, from a limited, and therefore incomplete, and possibly noisy or otherwise perturbed collection of already received signals. Thus, because the data set is incomplete and the capacity of the system is limited and the internal structure of the system is not arbitrarily flexible and adaptable, the system can only achieve some estimate *q* of this distribution. When the system receives now another signal *i*, it contains the subjective information

2 $$\begin{aligned} - \log q (i), \end{aligned}$$

while the information with regard to the distribution *p* is

3 $$\begin{aligned} - \log p (i). \end{aligned}$$

But since the signals are not yet known before they are received, the expected information gain resulting from the observation of a new signal is obtained by averaging these expressions over the probabilities of the individual signals. Although a rarer signal yields a larger gain of information, such a signal is observed less often. Therefore before one receives the signal, nevertheless one may still not expect a large gain in information, because with higher probability one has to expect that some more frequent signal occurs which then provides less new information. The expected subjective information gain then is

4 $$\begin{aligned} - \sum p (i) \log q (i), \end{aligned}$$

while the actual expected information gain from the distribution *p* is the Shannon information (1)

5 $$\begin{aligned} - \sum p (i) \log p (i). \end{aligned}$$

When *p* and *q* are different, that is, when the subjective estimate is different from the actual distribution, the latter is smaller than the former. Rather than being happy about the supposedly higher information gain due to the subjective hypothesis, the receiver should rather use the received signals to adapt its subjective distribution *q* and perhaps reduce the difference to the actual distribution *p*. In other situations, the receiver might want to obtain the best possible estimate *q* for the unknown *p* from a certain class of distributions. In general, the receiving system will not be able to model an arbitrary distribution, but its internal structure will restrict a priori the possible models. If the available class of models is fixed and the only issue is to determine the free parameters in this class so that the best possible *q* results, we are in the area of parametric statistics, an established and well studied mathematical theory.^9^ If the selection of the model class is an issue, e.g., to avoid overfitting, an over-adaptation to the signals previously received and their contingencies, one is lead to the field of statistical learning theory (Vapnik 1995, 1998). A fundamental result of this theory is that in order to obtain reliable error estimates, the number of received signals must be asymptotically larger than the number of degrees of freedom in the model.^10^ However, of course the number of degrees of freedom should not be too small either, in order to have a sufficiently large number of models available to capture the data fairly accurately. In the context of cognition, these principles are explored in Jost (2016). It may also happen that the received signals do not accurately follow the distribution *p* that one is searching for, but that they are perturbed or distorted by noise or other undesired effects. This too can be treated, in principle, within the framework of mathematical theories, but will not be pursued here.

Instead, we need to start a little deeper and face the fact that what we have taken as externally given data or signals, in essential respects are the results of constructions by the receiving system—which, incidentally, also makes the expression “receive” itself somewhat questionable.^11^ For a system, there are no information as such, no raw data, no input, but these result only from the insertion into an internally developed system of category structure. Categories allow the system specific and targeted observations. In this sense, information is generated as something important or relevant only by the receiving system. What does not fit into the category structure is not perceived by the system. What is without structure for the system, is random, is noise, and therefore automatically ignored. Only the categories allow the system to make distinctions. Thereby, a received signal becomes a carrier of information by reducing the uncertainty concerning specific measurements dictated by some category. However, the expected information depends not only on the category, but on the distribution *p* of the signals with respect to the categories. This distribution is not fully known to the system, but, as explained, the system can model it on the basis of the received signals by a hypothesis *q* and adjust this hypothesis continuously on the basis of the newly acquired signals. However, not only *q* but also the category system can be adapted and changed. An important difference from what we have said previously is, incidentally, that the probability distribution *p* is no longer independent of the receiving and observing system datum, but also depends on the category structure of the system (for a simple example, see Jost et al. 1997; this will be further analyzed in Jost 2017b).

The modeling by probability distributions *p* still falls short even despite this point, because the signals typically do not follow an independent distribution but have certain correlations, transition probabilities. Therefore, instead of a distribution *p* of signals, we should rather consider a stochastic process *X*.

The extraction of information means reducing uncertainty for the system. This uncertainty, however, can be reduced not only by observing the signal process *X*, but also by the identification of regularities in the process. Such regularities on one hand allow a compression of the data already collected for the purpose of efficient storage, and on the other hand they also provide a basis for predictions about future signals, i.e., a reduction of uncertainty, without these signals having to be actually received. Here, of course, it is assumed that the process *X* is stationary, that is, its statistical characteristics do not change over time.

Now comes an important point: regularities can only be found if a need for compression exists, as otherwise the received data can be reproduced in a completely faithful manner. A rule consists precisely in the best possible representation of the data under fixed constraints, i.e., internal restrictions. For such a selection to be made among the available options for the internal representation, the number of degrees of freedom has to be bounded, or, more precisely, there have to be fewer degrees of freedom than observations. Otherwise the need for compression is eliminated, and also what is contingent and follows no rule is shown in the data, if the data should be recorded as faithfully as possible. We are thus lead back under a new aspect to the central point of the statistical learning theory of Vapnik–Chervonenkis. In abstract terms, the finiteness and limitations of the cognitive system are a prerequisite for being able to make predictions with nontrivial content. (However, no system can fully grasp its environment by its own rules, since the latter as the more comprehensive system is necessarily more complex in terms of statistical physics. Because the system itself is part of its environment, in any case, we have to face the mathematical and philosophical problem of re-integration, self-reference.)

But the foregoing still underestimates the active component in the development of knowledge. To get this better into our view, we notice that the category of which we have spoken as a classification scheme for input signals is still a rather arbitrary formal principle. According to what criteria are categories formed, what determines the assignment of an input pattern to a category? Are categories rigid structures like drawers, into which inputs are sorted, or do they develop only through concrete experiences? In what sense there are general categories, are they not just collective labels for groups of exemplars? Or should we rather replace the diffuse and possibly abstract concept of a category with the more contentful one of the prototype? We are asking here for the organizational principles of knowledge, and we want, in particular, also cover the dynamical aspect inherent in this issue. In order to gain insight here, we analyze the concept of a gestalt. This term was introduced by von Ehrenfels (1980) as a psychological concept. With this term, he tried to explain the holistic identification of higher units, and important criteria for a gestalt were that it is more than the sum of its parts and that it is not altered by transpositions. Von Ehrenfels invoked here the example of a melody. We shall not discuss further the development of this concept as an organizing principle for perceptions, as a functional whole in the history of psychology. From the viewpoint of formal logic, a gestalt was conceived as an invariant under transpositions of a complex, or complementarily but equivalently, as an equivalence class under correspondences.^12^ This now clarifies the term formally and specifically reduces a gestalt to internal relations, but it still leaves open how the respective transpositions, correspondences, equivalences emerge and are selected. If there are no rules for this, a gestalt still remains a rather arbitrary ordering principle. This problem was solved in a joint work with Breidbach and Jost (2006). We took the transformation rules as a mathematical structure which the receiving system, which now becomes a perceiving subject, actively applies to its received input patterns. A gestalt consists therefore in the common characteristics of a set of patterns that can be mutually converted by transformations of a given type into one another, or in mathematical terms, as an equivalence class of the operation of a group of transformations, the invariance group of the gestalt. The new and crucial ingredient here is the formal structure of a group. A group is in this case within the meaning of mathematics understood as strictly here is a mathematically defined term; the main properties are that the elements of a group can be composed with one another and also be inverted, reversed, and that this operation is associative, that is, $A (BC) = (AB) C$, i.e., it does not matter whether we compose *A* with the result of the combination of *B* and *C*, or the combination of *A* and *B* with *C*. The order of the composition, however, in general makes a difference, i.e., *AB* may be different from *BA*. An illustrative example is the gestalt of a circle, which is independent of the location and size of a particular circle, so for the purposes of group theory, it is invariant under shifts and scalings. It is important that the groups that typically occur are finitely generated and presented, that is, they are already fully determined by the specification of finitely many elements and composition rules. Thus, we have a universal structure whose concrete realization is already determined by a finite number of parameters. We have thus obtained a general formal principle, with which a system can structure the input data of any kind for itself. In abstract terms, the set of transformation rules in turn follows general internal rules. These rules allow the system to reconstruct all the transformations that determine the gestalt from a few transformations between concrete available samples or prototypes. Of course, this can also be generalized by employing other algebraic structures. This is for instance important for the formal transformation grammar in linguistics. In any case, this approach will allow us—or the system we are considering—to generate a gestalt from a set of patterns that have certain similarities by a transformation group generated from those similarities. We only need to find transformations which convert the patterns into one another, and take those as the generating elements of a transformation group. The resulting gestalt will then contain even more, new pattern, namely all those that arise from the application by the group elements on the initial patterns. The gestalt of a circle can therefore be generated by specifying 2 or 3 circles of different size and location. A gestalt is thus determined by the specification of a few representatives and the associated transformation group. Larger groups correspond to more general gestalts, and inclusion relations between groups can be translated into a gestalt hierarchy. The larger the group, the less properties are left invariant by all group elements. To quantify differences between patterns or representatives belonging to a gestalt, we have to introduce an additional structure, that of a norm^13^ of a transformation, which measures the size of a transformation that is required to convert one pattern into another. Because in general there are several ways to transform a pattern to another, in a specific situation, we encounter the optimization problem of finding a minimum norm transformation between two given representatives of a gestalt. An example from psychology of perception is the reaction time required to identify a rotated pattern with a prototype. It turns out that this reaction time is proportional to the angle of rotation, the obvious norm of a rotation.

If such norms, that is, measures of the magnitude, or in another interpretation perhaps also the cost of executing a transformation, are given or obtained, we can also construct a prototype for a gestalt, as a member of the gestalt with the smallest average distance from the other members. Again, the prototype need not occur as a specific pattern in the available input, but it is a construction of the system on the basis of general transformation rules utilized by it. Even such a prototype need not be explicitly represented in the system, but it is enough to have the implicit possibility of making distance comparisons between members of a gestalt. If now conversely the system is provided with a specific input pattern, it can determine whether it belongs to one of the gestalts constructed by it. For this, in principle, there are two possibilities:

1. Transformation of the pattern by an element of the group defining the pattern into a prototype that has been constructed as described above. Here, the system will try again to achieve this via a transformation of smallest possible norm, in order to have at the same time a criterion for how well the pattern matches to the gestalt, in particular whether it is central or rather marginal. It will be helpful to first submit the pattern to a certain normalization to reduce the transformation possibilities that need to be checked. Geometric figures can for example be rescaled to a standard size, they can be moved so that their center of gravity is in a fixed position, etc.
2. Evaluation of the invariants characterizing the gestalt. These are typically internal relations, such as pitch differences between successive notes in a melody, rhythmic relationships, relative position of the points of a geometric figure to each other, or more abstract quantities such as eigenvalues, etc. These invariants could be obtained implicitly also by a kind of statistical inference when the system learns similarity relations.

In practice, probably hybrids of these methods will be most effective. Some results in this direction are known from perceptual psychology experiments. For instance, Smith and Minda (2002), subjects decide on the membership of a visually presented pattern to a gestalt based on the similarity to the prototype—which is only constructed within or from the gestalt itself—rather than by comparison with the concrete sample copies provided to the subjects. It would be insightful to have further studies to determine to what extent the investigation and evaluation of invariants is used in perception as a criterion for gestalt membership.

By means of active transformation rules from (for example, sensory) input patterns a gestalt is thus generated that captures what is common to these patterns, what is invariant. These rules are general and in particular intersubjectively valid; the individual generates them from the interaction of genetic predispositions, cultural socialization mechanisms, internal self-organization processes set in motion by external impulses and individual learning processes building upon that. The individuals obtain their knowledge and thereby constitute themselves in the interplay between its endowments and its morphogenetic principles on the one hand and the active sensorimotor exchange with its environment and interactions with other individuals that may get intensified by resonances on the other hand.

This is particularly evident in learning the mother tongue. The Chomskyan reference to the stimulus poverty, that is, the underdetermination of linguistic structures by the speech examples available during the child’s learning process, has exposed a fundamental error of behaviorism, which tried to explain the child’s learning of the mother tongue by simple stimulus-response schemes. Chomsky (1959) has probably eliminated behaviorism as a serious theoretical approach. For learning language, one needs an internal structure of the type of transformation rules of the universal grammar of Chomsky (see Chomsky 1965, 1981, 1995 for the various stages of the development of Chomsky’s theory) or something similar. Such a structure, however, could also, in combination with the other components mentioned above, emerge in a self-organization process and would only have to be implicitly represented in the system, as postulated by connectionism, rather than being genetically fixed and (more or less) explicitly (but not necessarily consciously) given, as Chomsky thinks. But this is not yet decided, and perhaps we need a conceptually deeper approach. We should not forget that in expressions of everyday language, the grammatical transformations are apparently so tightly interwoven with semantic relationships and implicit references to the context, which are understood by the listener that automatic language interpretation and translation programs for the present state of theoretical linguistics are still out of reach,^14^ and also the connectionist models quickly reach their limits. In particular, the linguistic knowledge incorporates and integrates grammatical, semantic and contextual components in a complex manner. In Tomasello (2003), Tomasello argues against Chomsky’s paradigm for a usage-based approach, emphasizing that human children have more powerful learning mechanism at their disposal than simple association and induction. They can infer the intentions of other humans, and they possess sophisticated skills of pattern finding. The ability of pattern finding is, of course, one of the central themes of the present essay.

## Knowledge and cognition

So far, we have analyzed how knowledge is created and comes about, and we have found that this process is not just a passive data storage process but requires active use of internally given structuring rules. The generality and universality of these rules then makes intersubjective compatibility of acquired and generated knowledge possible.

This knowledge then allows the subject to deal with new experiences, by relating them to previous ones and thus making them potentially meaningful. Knowledge as memory allows us to compare the new with the already known. Conversely, knowledge makes past experiences currently available by capturing them in an internal schema. In its knowledge, the subject knows statistical and other regularities of the input world and in particular the code that decodes or decyphers the information contained in specific inputs.

Now there are different kinds of knowledge, and a more detailed analysis provides insights into the process of creation and the structure of knowledge. Because, as argued, knowledge is not just an unstructured storage of data, of so-called “facts”, but also in particular represents an organizational principle, there follows directly a distinction between factual knowledge or expertise on the one hand and structural, storage or organizational knowledge on the other. The internal structure rearranges the data and by its internal classification leads to a higher efficiency and lower storage costs, but then also requires knowledge of this internal organizational structure itself. One no longer remembers the data themselves, but where and how to find them. In the knowledge organization we thus find the transition from content to form. This is also related to the transfer of the complexity from the direct relationship with the outside world to the internal organizational structure (see Jost 2004). This then leads to the formal consideration of the question of the optimal knowledge representation.

If at this point already a short leap is permitted from individual knowledge to cultural and social knowledge, we see whole knowledge or scientific fields that focus for the most part only on such structural knowledge, such as jurisprudence or computer science. One does not learn the civil law by heart, but rather the method to find the relevant laws and legal judgments in a specific case. Similarly, computer science is concerned with the general principles of data organization and storage. For an analysis of how every action, every concept and even every experience of the individuals in a society depends on how the culture of that society understands and structures knowledge, we refer to Neuser (2013).

Another distinction that is important for cognition, which is transverse to the above distinction, is that between declarative and procedural knowledge, that is, the distinction between content and process knowledge, between the “what” and the “how”. This is also the distinction between explicit and implicit, tacit knowledge, between knowledge which can be explicitly formulated and recalled in isolation, and knowledge which is only implicit and is utilized only in the flow of a process. In particular, knowledge is not necessarily aware of itself.

## Self-reassurance of knowledge

However, more is at stake. The process of knowledge creation consists not only in the inclusion of inputs into an organizational structure and the adaptation of this structure to structures to be detected in the inputs—and the double occurrence of the word “structure” already hints at a difficulty—but also in the revision of this structure according to the experience of the outside world through the system. Knowledge has no independent authority in itself by which it can control itself, it need not even necessarily know about itself, and it can gauge itself solely by its source, which in the end is the outside world. On the basis of its knowledge, the system can act purposefully, but this action carries no guarantee of success in itself, but may fail. Incorrect knowledge of the chairs in the room, the knee hit in the dark. Plausible, but unfortunately too simplistic.

The system knows nothing of the outside world than just its knowledge. Only through the artifice or sleight of hand that we postulate us as external observers of the system in its environment, we can gain a concept of an independent reality at all, but this is unavailable to the system itself. We seem to fall, therefore, into a dilemma. From this perspective, the system has neither in itself nor outside of it a controlling instance. The difficulties dissolve only when we make a temporal differentiation and conceive at the same time knowledge creation as a double feedback process. Knowledge is not there, but is formed, and in this formation process the overwhelmingly large, in principle available data set is not completely stored, but on the basis of the internal structure significant aspects will be singled out selectively and have their compatibility with this structure checked. This is the inner feedback loop. The outer loop generates new data through action which is specifically directed by the already existing and developed structures.

The above is the system theoretical trick of resolution of a structure in a process, the emphasis on the preliminary and unfinished. While this is important, and we will come back to it, it evades the question raised here. There are other approaches:

1. The reflection of knowledge about itself. While this is not entirely possible, since the reflection on the reflection on the reflection ...results in the well-known endless recursion, and must therefore always omit aspects about itself, but formally of course, a part of a system can separate itself as an though imperfect observer of the rest of the system. This self-reflection of knowledge as formulated in the Socratic proposition “I know that I know nothing” can therefore be considered as the beginning of Western philosophy. Now this sentence is formulated negatively, but from this insight fundamental issues of the possibility of knowledge can then be analyzed. This leads into the history of philosophy, but should not be further illuminated here from the perspective of the history of philosophy.
2. The realization that there are other, similar knowledge carriers whose knowledge one can acquire. You therefore no longer need to always take the trouble to acquire one’s knowledge by oneself—the difficulties of internal structuring of inputs as a basis of knowledge have been pointed out above—but you can get knowledge as already structured condensed experience of others. This is not automatically possible, of course, but requires first the ability to consider other knowledge carriers not only as parts of the external world, but to recognize them as systems that are of the same kind as oneself. For humans, the prerequisite for this is the ability of empathy, to be able to empathize with others, in order to exploit how they deal with problems with which one will also be confronted oneself. Likewise, one can mitigate the unpredictability of the others by assuming that they are similarly constituted as oneself, with similar feelings and desires, and therefore can also be expected to behave similarly as one would do oneself. This is certainly not wrongly emphasized in modern anthropology, in particular in the work of Tomasello (1999). If this capability is available, also internally compiled knowledge structures can be transmitted through communication. The importance of communication is then obvious and therefore need not be further elaborated here. Knowledge becomes the knowledge of a community by individuals preserving individual aspects of knowledge and making them available as needed and also taking care of their preservation and tradition. See for instance the analysis in Neuser (2013).
3. The external storage of knowledge. This requires a symbolic representation of knowledge and leads from the invention of writing to the modern databases. The knowledge of the outside world is thus stored back in the outside world. Thus, the boundaries between the data and their representation get blurred. The book does not need to remain a representation of something else, but can become a separate object of knowledge, namely a more highly structured one than the object area to which it refers. By the fact that the outside world can now include representations about itself, it will become at the same time more regular and more complex for the epistemic system and thereby in turn grants the system a growth in complexity.

## Representations of knowledge

So far we have treated knowledge implicitly as individual knowledge, as the knowledge of an individual. But by items (2) and (3) of the preceding section, there is also cultural, social, technical, ...knowledge that is available to all members of a community and that is preserved as a common tradition or stored in books, databases, .... We can now understand this common knowledge not only as an extension of individual knowledge, but also highlight different ways of structuring. A comparison should therefore be illuminating.

Here we can distinguish three systematic issues, namely those about

1. Storage: where is the knowledge?
2. Code: how is the knowledge represented?
3. Access and organization: how and where to find specific knowledge?

These questions are obvious, and in many (but not all) situations, the answers are just as obvious. The computer science represents content traditionally in the code that is simplest and therefore considered to be fundamental, the binary one, and it stores data explicitly as a binary sequences on the hard disk or other storage media and uses concepts such as stack and queue for assembly and, for example, hierarchical search trees for access. Some alternatives can be found in the various realizations of the concept of the encyclopedia as an arrangement of all the available knowledge and of a—more or less successful—solution of the problem of specific search.^15^

- In Western antiquity, from the conception of Aristotle of a systematic presentation of all available knowledge in individual tracts a hierarchical organization emerges as superimposing structuring principle which is developed in Hellenistic library catalogs.
- The ancient Chinese encyclopedias represent the most extensive and most ambitious attempt ever undertaken of systematic arrangements of the entire knowledge. It involves a systematic organization of knowledge available in one linear sequence immanently dictated by the content, which, however, was partly based on more extraneous combinatorial schemes rooted in general cosmic ordering principles.^16^ Some of the classification schemes in these encyclopedias appear rather arbitrary and not necessarily appropriate for the subject matter from the perspective of contemporary science which is based on causal rather than systematic principles. For example, the turtle is treated as a fish because of its aquatic life.
- Incidentally, the Chinese encyclopedias do not contain an index. Index and table of contents as a formal tool for accessing specific knowledge from a source were introduced by the schoolmen in Europe in the thirteenth century.
- Associative knowledge networks: the related concepts of Alsted, Kircher (see Kircher (1669)) and others regain actuality, as emphasized by Breidbach (2005); Breidbach (2007); Breidbach and Ghiselin (2006). Here, too, of course, many details can be criticized as in the Chinese encyclopedias. For example, the ideas of Kircher for the deciphering of Egyptian hieroglyphics nowadays seem abstruse, although they become understandable within his system. These knowledge networks can also be seen in the tradition of mnemonic systems (Yates 1966; Rossi 1983) which became elaborate representations of an assumed structure of the world. The Jesuit missionary Matteo Ricci was inspired by such ideas to his “memory palace”, a method for memorizing the Chinese characters by associatively linking them with visual imagery, by positioning them in an imaginary spatial setting (for a short description and other supplementary bibliographic references see Spence 1984). Our memory cannot keep unstructured data well, and the method of Ricci represents an original approach to represent a largely unstructured^17^ dataset by superimposing an internal organizational principle that is extrinsic to the data.
- The internal logic of knowledge: deduction of knowledge from a single principle: this is the approach of Leibniz, which thus provides an explanation for the coherence of the world and of knowledge. In particular, this requires for Leibniz the replacement of associative networks by a more rigorous formalism, which for him is based on combinatorial rules.
- European encyclopedias as arrangement of knowledge in individual articles in alphabetical sequence with a system of cross-references. A systematic, associative or logical arrangement is replaced by one based on a convention (the Latin alphabet). As a search tree, the latter allows to quickly find entries based on their spelling; in a Chinese dictionary, this is much more complicated and cumbersome. On this, the system of cross-references is superposed that produces associative links based on content relationships.
- Encyclopedias on CD-Roms: the actual ordering principle is not disclosed to the user, who can acquire only knowledge through cross-references (links). Anyway, the electronic storage makes a hierarchical rather than a linear organization of knowledge possible.
- Databases are limited to a fixed subject area and want to develop the knowledge available for that field with the help of interactive decentralized inputs. This results in both the usual questions and problems of organizational structure (in particular fast access, efficient storage, easy changeability of entries (updates)) and automated data collection as well as those of the internal coherence of the various items. More problems arise when one wants to link databases together, because the respective organizational principles are usually not readily compatible. One must therefore create metastructures, in which the individual structures can find their places.
- Computer networks: upon entering a search entry, search engines systematically search Internet sites according to a sophisticated mathematical search procedure that utilizes superficial word similarities and links between websites. The intention is to make all existing (on the Internet) knowledge about the chosen keyword available to the user. By a simple Google search, an answer can be obtained on any issue, but the quality of the proposed answers cannot be guaranteed. The user does not usually have the resources to check the facts claimed on the websites identified by the search engine. This entails the risk that the independent, critical and creative aspect of knowledge acquisition, selection and production is lost. This risk must be addressed through the development of new methods of source criticism. We are also led back to the problem of finding certainty of knowledge, now, however, no longer as a problem of internal consistency and coherence and the external reference of individual knowledge, but also as a social challenge of information selection and evaluation. It may seem that thus another old problem, namely the availability and acquisition of information for individuals is converted into its opposite as information overload. According to our analysis, this is only apparently so, because the importance of information is only revealed in an internally structured context, and the task to condense meaningful knowledge from a flood of data cannot simply be solved by increasing that flood of data. Only meaningful prestructuring of these data might provide help (without relieving the individual of its own independent critical evaluation), but precisely here lies the difficulty.

All featured encyclopedic concepts show in one way or another a predominance of the systematically ordering over the logical or causally analyzing thinking, and they also each reflect the intellectual trends of the eras that created them, and perhaps in turn contributed to shaping those trends.

But how does this compilation of different methods to represent knowledge and make it available as systematically and completely as possible help us? All these methods have their shortcomings. The problem appears to lie in the explicit form of storage. Our brain does not work like an encyclopedia or a database nor like the Internet. So far, the human head is smarter than all of its products. But how then does it represent this knowledge? And to what extent can we speak here of “representation”?^18^

Certainly, to move forward, we need to turn away from the idea of a simple facts memory. A genome contains knowledge about the environment, but not as retrievable facts, but as a guide for the production of proteins—and at the same time as a guide for the manufacturing process itself—that form cellular structures and processes, which in turn constitute an organism that has a chance to compete in its environment and replicate. The storage as such is indeed explicit, as a chain of molecular building blocks, the nucleotides, but there is no direct reference to any environmental data, for example, the temperature of the habitat or the possible food sources. The genome encodes only the development scheme for an organism, a structure that can carry out its metabolic processes in its habitat, or, in another perspective, it contains those structuring rules that can maintain an autopoietic process with the ingredients available from the environment. The genome however knows nothing about that. Neither does the organism (see Jost 2017a for further discussion of this issue).

However, this leads us to an important aspect, namely that of the *complementarity of knowledge*.

The organism does not need to know, for example, that a certain plant contains important nutrients for it. It just needs to feel the hunger to eat this plant, and to know where to find such a plant. And if this plant grows throughout its habitat, it does not even need to know that. The organism therefore does not need to know the structural regularities of the environment, but only needs to know how to exploit them, if knowledge is required at all for that. It does not need to know the obvious, the automatically given, and it cannot know what is accidental or totally irregular (although from the standpoint of cognition, causality is reversed here, because it is precisely what a system does not know and does not understand which is randomly and arbitrary for it). Its knowledge operates between these extremes. Now we no longer have two components, but three, namely environment, genome and organism. From the perspective that has been implicitly selected here, the environment does not know anything, but has only certain imperfect regularities. These regularities are a prerequisite for the existence of the genome. The genome has just been selected because, on the basis of these regularities and possibly also by favorable accidents that have enabled it to exploit further regularities, it has been able to spawn organisms that have been able to discover more regularities. The genome has thus learned these regularities in the course of its evolution; for the organism, they are then simply given, and it just needs to know how to exploit them. Again, and this is an important point for our purposes, knowledge requires an internal structure, which condenses experiences acquired on a different time scale and makes them expectable as regularities. The latter are nowhere represented, but their presence makes their representation unnecessary. This is the complementarity of knowledge.

So what is there between these two extreme poles, the encyclopedia, in which all the knowledge available is stored in a form that enables an explicit, direct access, and the genome, in which only production rules are encoded for the ingredients for building and maintaining an internal process which then can achieve its own replication, i.e., between—at least in intention—a complete representation and the absence of the need for any representation?

It may seem helpful at this point to return to the distinction between explicit and implicit knowledge. Instead, we now want to blur this distinction. For that purpose, we consider the so-called connectionist approach of cognition research. Its basic model is the neural network, a dynamical system whose internal attractors represent the response capabilities of the system to external inputs. The input is in this case the initial condition for a dynamical iteration, which evolves over time from a transition phase into an invariant stable inner activity sequence. “Invariant” means here that this sequence is repeated periodically. In the simplest case it is a fixed point, that is, the dynamics comes completely to rest at a certain intrinsic value. Another possibility is a periodic orbit, which is always traversed in the same way. However, there may also exist chaotic attractors, which while compressing the dynamics, still can internally diverge. “Stable” means here that the system returns to the same attractor after a not too large perturbation. A dynamical system typically has many and different attractors, and which is realized depends on the initial conditions. In this sense, therefore, the attractors classify the initial conditions, that is, in the situation under consideration the inputs.^19^ Each attractor has its own basin of attraction, and when an input in falls into this range, the dynamics will approach the corresponding attractor. In this manner, an input classification is possible, in which similar inputs typically lead to the same attractor, while more distinct one can run towards different attractors. Now, however, the attractors in a way are only virtual states, because they will only be realized if the appropriate input comes. They represent the dynamical possibilities of the system. The system of attractors of a dynamical system is determined by the system parameters, in particular through the coupling strengths between its various elements or parts. This coupling strengths or other system parameters are usually concretely physically realized. In a neural network, these are the strengths of synaptic connections between individual neurons. A particular such parameters, however, cannot be assigned to a single attractor, but exerts an influence on the dynamical capabilities of the system as a whole. This structure of possible dynamical time courses can be influenced by varying those parameters. If one wants to make a particular input to run into a given attractor, one has to adjust the system parameters accordingly. If this is done systematically, one speaks in this context of learning.^20^ But then, learning is a global and distributed process in the system, even if the correspondence between inputs and attractors to be produced is local and concrete. On the one hand, many parameters have to be adjusted, if perhaps only slightly, and on the other hand, the adjustment of each parameter has an impact on the whole dynamics, and thus also on the dynamics set in motion by other inputs. Thus, the system represents its input classes on one hand virtually through its dynamical capabilities, on the other hand in a distributed manner in real physical parameters that simultaneously reflect the influences of all input classes.

## Neuronal and cultural knowledge

In the preceding section, we have also seen an interaction between different structures that contain, represent, or process knowledge. In his monograph (Breidbach 2013), Olaf Breidbach has analyzed the interaction between the brain and the culture, and he has coined the term of “neuronal aesthetics”. Here, “aesthetics” does not refer to the concept of beauty, but more generally to its origin in the Greek word 
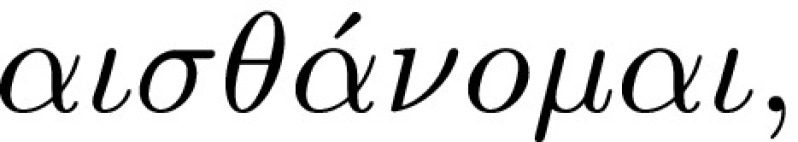
 to sense.

Both the neuronal and the cultural system constitute a dynamic web of relations within which facts acquire their meaning and relevance and for which external stimuli come about as perturbations of their internal dynamics. In either system, the external world does not enter as a direct representation, but is only indirectly constructed from the internal perspective that records and evaluates those perturbations. External stimuli have the effect of making the internal dynamics more specific. They canalize the dynamics so that it gets into a position to explore more specific options, and it is precisely this what leads to the evolution of such systems. As explained above, the interplay of external stimuli and internal structures and dynamics can reduce complexity and thereby enable the system, be it a neuronal or a cultural one, to acquire new forms of complexity. Here, as in biological evolution, each system is constrained by its own structure, but in turn, this structure can also canalize the system towards new evolutionary possibilities (see the discussion in Jost 2017c).

The interaction of these systems offers evolutionary potential in both directions. In one direction, cultural traditions obviously provide knowledge for individuals and shape cognition their cognition and thereby offer a potential for accumulation that cannot be achieved by individuals without communication. In the other direction, beyond the trivial fact that culture emerges from interactions between individuals, externalization of cognitive techniques, as we saw in the preceding section, triggers cultural dynamics. The interaction opens each system for new evolutionary possibilities. Either type of system can benefit from complexity gains of the other.

In order to understand knowledge, we therefore need to analyze the interaction and the relations, or in mathematical terminology the morphisms (see Jost 2015a), between these two webs of relations. They are not isomorphic, and they have developed different schemes of codings, they operate on different time scales, they are rigid or flexible in different ways, but they can relate to each other and interact. In fact, much of their structure and dynamics is derived from and dependent upon their interaction. Olaf Breidbach had hoped to develop a morpho-logic to capture these morphisms between experience and physiology without the pitfall of trying to reduce one to the other, in order to gain deeper insight into both of them. This is his program of a neuronal aesthetics.

## Knowledge and process

In this last section, we summarize some of our insights in an abstract manner and provide an outlook that links our analysis to recent technological advances and prospects.

Knowledge is by its nature something static and thus cannot be directly conceived as a process, but only as a result or a condensate of a process. Conversely knowledge structures cognitive processes. This interplay can be only captured in the interaction of two different time scales, a slow one, on which the knowledge is acquired, learned, produced and arranged, and thereby changed, and a fast one, on which it is used structurally to process, organize, evaluate and also systematically generate incoming data and signals. In this perspective, the Shannon information theory operates on the fast, the statistical learning theory on the slow time scale. Thus, we do not have here competing theories, but ones that can complement and support each other. To make this fruitful, an integration of the two time scales is required. This then also resolves the confrontation, an essentially static perspective, of structure and process, and transforms it into a dynamic interaction. That something like that is required is already shown by the simple consideration that on the fast time scale, the system parameters that incorporate the knowledge of the system are fixed and the input, for instance, the sensory signals, changes while on the slow scale the system parameters are adapted, hence changed, whereas the signal distributions, or more generally, the regularities underlying the input, in contrast are assumed to be fixed. In fact, these regularities show up only on the slow scale because only by the observation of many data, on the one hand random fluctuations average out and on the other hand more complex laws can be detected.

Thus, as shown in the above analysis, we have the mutually coupled processes of knowledge acquisition and knowledge structuring. In some situations, these processes can be implemented and controlled on purpose, such as when building a database. Deeper insights can be probably obtained from processes in which the structure emerges from a collective, distributed dynamics of mutually coupled but still partially independent units, i.e., by a so-called self-organization process. In particular, the artificial neural networks already mentioned have been introduced to implement this. The central idea of this theory is that synaptic connections between the individual elements, the (formal) neurons, get strengthened according to the correlation between the activities of the two neurons involved.^21^ However, this leads to the problem that those synapses then continue to strengthen, and one has to compensate for this effect by a forgetting mechanism. A mechanism that is theoretically elegant (Gerstner et al. 1996) and well supported in neurobiological experiments (Markram et al. 1997) is the so-called spike-time-dependent synaptic plasticity that depends on the temporal relationships between the dynamics of the neurons involved (this is also compatible with the original formulation of Hebb’s rule). Here in analogy to brain cells, the formal neurons sum the incoming excitations received by them via synapses from other neurons, until a firing threshold is reached, and then produce an excitation pulse that is propagated to other neurons via synaptic connections again. These synapses are directed, that is, let excitations through in one direction only, and one can therefore distinguish the presynaptic and postsynaptic neuron in each constellation. The mentioned learning rule now increases the strength of the synapse when the presynaptic neuron fires shortly before the postsynaptic one, that is, when the incoming excitation contributes to activating the postsynaptic neuron. On the other hand, the synapse is weakened if the temporal order is reversed (see Jost 2006 for a mathematical model).

By means of such learning rules associative networks can be built that can associate an input to a stored pattern. In the above-mentioned context of dynamical systems, each input class can be translated into a specific dynamical activity, which then just represents the stored pattern to which the input belongs. By a stored pattern, one would initially mean something imposed from the outside, but more interesting are internally evolving and emerging patterns. Now through the operation of the relevant learning rules, when exposed systematic inputs actually internal attractors develop in the network, which then classify the inputs and thus assign them to internally constructed representations. However, the question is how far this approach really carries, in particular, whether through such a simple, purely local learning rules also higher structures, such as the gestalts discussed above can emerge. Perhaps it is more likely that such association rules can be useful only within a process that is set in motion and maintained by more general structural principles.

The application of neural networks has recently seen dramatic advances, by creating so-called deep neural networks, that is, networks, that like the mammalian neocortex, contain several layers. In fact, instead of only six layers, as we possess them, these networks often derive their performance from hundreds of layers. Perhaps the need for so many layers indicates that those deep neural networks capture one important aspect of how the mammalian, and in particular, the human brain functions, but other key aspects probably still elude them. Better understanding those principles of brain function should be expected to lead to further dramatic improvements of the performance of artificial neural networks.

In machine learning, a somewhat different approach is pursued. One starts with certain structural priors to handle high dimensional data sets. For instance, in compressed sensing (Candès et al. 2006; Donoho 2006), one assumes that there only exist few sources that have produced those high dimensional data. In manifold learning (Belkin and Niyogi 2003), one assumes that the data sit on or near an intrinsically low dimensional smooth manifold, which may then possibly stretch in a complicated manner into the high dimensional data space. Or one assumes that the data are intrinsically sums, with only a few terms, of products of low dimensional vectors (Hackbusch 2012). A question is whether these approaches can be subsumed under more general principles, in light of the preceding considerations. This is further discussed in Jost (2016, 2015b).

## Footnotes

1 So the title of a popular science book of physicist Weinberg (1993).

2 For details, see for instance Jost (2005), 8.1, and the references given there. Instructive computer simulations of cellular automata can be found at http://www.ddlab.com.

3 That knowledge about knowledge is possible will also guide some of our subsequent considerations.

4 This is also the basis of Haken (1990).

5 The concept of an icon, however, is not without problems, see Eco (2002) p. 200ff.

6 For a more detailed treatment of some aspects of the sequel, see Jost (2004).

7 This is somewhat analogous to a discussion in economic theory, the concept of the price as labor value vs. market balance between supply and demand. In the economic context, the value of information can be measured by the price that the receiver is willing to pay for the receipt of this information Arrow (1971). The value of the information thus does not lie in the difficulty of obtaining it or in its content expressible in bits, but in benefits for the receiver as reflected in the price that can be obtained. The monetary code in this respect is better adaptable than the information-theoretic bit code to the total internal state of the receiver; the latter takes into account only the current state of knowledge of the receiver and measures its increase through the information. For a systematic treatment in the context of game theory, see Bertschinger et al. (2015).

8 Important lessons can be learned here from the insight of Von Uexküll (2014); for a formal approach in this direction, see for instance Ay and Löhr (2015).

9 The competing Bayesian approach to statistics is based on prior probabilities for each model and adjusts them based on the probabilities with which the models postulate the received signals. The conceptual and decisive problem that decides about the success in a concrete situation is whether or in a specific situation how well one can construct those prior probabilities. The current trend in many disciplines favors the Bayesian over the parametric approach. The Bayesian scheme is computationally more expensive than parametric statistics, because entire probability distributions have to be computed instead of single parameters, but the ever increasing available computer power makes this less relevant.

10 As measured by the Vapnik–Chervonenkis dimension.

11 This also has quite problematic aspects. If knowledge is no longer “objective”, but a subjective adjustment placing data in an internal dynamic process, that is, when knowledge is only constructed by the interpretative performance of the subject, the objectivity of observer testimonies or historical and other sources becomes questionable. See Fried (2012) for an analysis of this issue, and for an instructive case study, see Fried (2003).

12 See Grelling and Oppenheim (1988); I am grateful to B. Smith for this reference.

13 “Norm”, like “group”, is understood here as a strictly defined mathematical concept; therefore colloquial connotations lead the understanding only astray. The norm of a transformation is a measure of the difference from the so-called neutral or trivial transformation that changes nothing, but leaves everything as it is. For a rigid translation, the norm is given by the distance between the original position and that caused by the translation. The norm of a rotation can be measured by the rotation angle.

14 I had tried to use Google translate for translating my original German text into English, but many sentences and phrases came out completely garbled. In particular, such automatic translation schemes which are based on *n*-grams, with $n=5$ typically, are not capable of detecting long-range connections, as in German split verbs.

15 These issues have been discussed in detail by Olaf Breidbach.

16 The systematic organizational structures of Chinese encyclopedias have apparently not yet been systematically studied in the literature. For an overview of the historical development see Schmidt-Glintzer (1990), p. 308ff. The intellectual background of the encyclopedists is represented in Needham (2017). For a satirical treatment, see Borges et al. (1942).

17 I don’t intend to deny here that underlying the Chinese characters there is a complex systematics that is based on both phonetic and conceptual relations, which goes far beyond the pictorial element which is usually naively considered as being the essential principle. But this becomes evident only to the educated literates as the fruit of long studies.

18 See the discussion in Ziemke and Breidbach (1996).

19 For the construction of the classification system see also the above remarks on the gestalt concept.

20 This is dealt with in the already mentioned statistical learning theory and the theory of neural networks, see e.g., Jost (2017b).

21 This is the Hebbian theory, named after Hebb (1949). Hebb himself traced this rule, however, back to Sigmund Freud. But this is still not the original source, since this rule had been formulated within a conception of an associative network already by Simon Exner in 1894 (Exner (1999)), see Breidbach (2001).

## References

[CR1] Arrow KJ, McGuire CB, Radner R (1971). The value of and demand for information. Decision and organization.

[CR2] Ay N, Bernigau H, Der R, Prokopenko M (2012). Information-driven self-organization: the dynamical system approach to autonomous robot behavior. Theory Biosci.

[CR3] Ay N, Löhr W (2015). The Umwelt of an embodied agent—a measure-theoretic definition. Theory Biosci.

[CR4] Bateson G (1972) Steps to an ecology of mind. In: Collected essays in anthropology, psychiatry, evolution and epistemology. Chandler Publishing Company

[CR5] Belkin M, Niyogi P (2003). Laplacian eigenmaps for dimensionality reduction and data representation. Neural Comput.

[CR6] Bertschinger N, Wolpert D, Olbrich E, Jost J (2015) Value of information in noncooperative games. arXiv:1401.0001

[CR7] Borges JL (1942) El idioma analítico de John Wilkins, La Nación, Argentina. Reprinted in: Borges JL (1996) Obras completas, Vol. II. Edecé Editores, Buenos Aires, 1976, pp 84–87

[CR8] Breidbach O (2001) Hirn und Bewusstsein—Überlegungen zu einer Geschichte der Neurowissenschaften. In: Pauen M, Roth G (eds) Neurowissenschaften und Philosophie. Eine Einführung. W.Fink Verlag, München, pp 11-58

[CR9] Breidbach O (2001). Deutungen. Zur philosophischen Dimension der internen Repräsentation.

[CR10] Breidbach O (2003) Zur Repräsentation des Wissens bei Athanasius Kircher. In: Schramm H, Schwarte L, Lazardzig J (eds) Kunstkammer, Laboratorium, Bühne. Schauplätze des Wissens im 17. Jahrhundert. Walter de Gruyter, Berlin, pp 282–302. English version: Breidbach O (2005) On the representation of knowledge in Athanasius Kircher. In: Schramm H, Schwarte L, Lanzardig J (eds) Collection, laboratory, theater. Scenes of Knowledge in the 17th Century, vol. 1. Walter de Gruyter, Berlin, pp 63–76

[CR11] Breidbach O (2005). Bilder des Wissens. Zur Kulturgeschichte der wissenschaftlichen Wahrnehmung.

[CR12] Breidbach O, Vercellone F, Bertinetto A (2007). Rappresentazioni del sapere. Un confronto delle forme di rappresentazione della scienza universale barocca (Athanasius Kircher) con quelle della modernità. Athanasius Kircher. L’idea di scienza universale.

[CR13] Breidbach O (2008). Neue Wissensordnungen. Wie aus Informationen und Nachrichten kulturelles Wissen entsteht.

[CR14] Breidbach O (2011). Radikale Historisierung. Kulturelle Selbstversicherung im Postdarwinismus.

[CR15] Breidbach O (2013). Neuronale Ästhetik. Zur Morpho-Logik des Anschauens.

[CR16] Breidbach O, Ghiselin M (2006). Athanasius 
Kircher (1602–1680) on Noahs Ark: Baroque “Intelligent Design” Theory. Proc Calif Acad Sci.

[CR17] Breidbach O, Jost J (2006). On the gestalt concept. Theory Biosci.

[CR18] Breidbach O, Kutsch W (1990). Structural homology of identified motoneurons in larval and adult stages of hemi- and holometabolous insects. J Comp Neurol.

[CR19] Breidbach O, Vercellone F (2011). Anschauung denken.

[CR20] Candès E, Romberg J, Tao T (2006). Stable signal recovery from incomplete and inaccurate measurements. Commun Pure Appl Math.

[CR21] Chomsky N (1959). Review of verbal behavior by B.F.Skinner. Language.

[CR22] Chomsky N (1965). Aspects of the theory of syntax.

[CR23] Chomsky N (1981). Lectures on government and binding.

[CR24] Chomsky N (1995). The minimalist program.

[CR25] Donoho D (2006). Compressed sensing. IEEE Trans Inform Theory.

[CR26] Eco U (2002). Einfhrung in die Semiotik.

[CR27] Engel A, Friston K, Kragic D (2015). The pragmatic turn. Toward action-oriented views in cognitive science.

[CR28] Exner S (1999) Entwurf zu einer physiologischen Erklärung der psychischen Erscheinungen, Frankfurt. Reprint of the original 1894 edition

[CR29] Fried J (2003) Geschichte und Gehirn. Irritationen der Geschichtswissenschaft durch Gedächtniskritik. Abh. Geistes- und Sozialwiss.Kl. d. Akad. Wiss.Lit. Mainz, Jg.2003, No. 7

[CR30] Fried J (2012). Der Schleier der Erinnerung: Grundzüge einer historischen Memorik.

[CR31] Gerstner W, Kempter R, van Hemmen J, Wagner H (1996). A neuronal learning rule for sub-millisecond temporal coding. Nature.

[CR32] Grelling K, Oppenheim P (1988) Der Gestaltbegriff im Lichte der neuen Logik, Erkenntnis 7, 1937/38, pp 211–25. English translation: The concept of Gestalt in the light of modern logic. In: Smith B (ed) Foundations of Gestalt Theory. Philosophia Verlag, München, pp 191–205

[CR33] Hackbusch W (2012). Tensor spaces and numerical tensor calculus.

[CR34] Haken H (1990) Synergetik, 3rd edn. Springer, Berlin

[CR35] Hebb D (1949) The organization of behavior, New York

[CR37] Jost J (2004). External and internal complexity of complex adaptive systems. Theory Biosci.

[CR38] Jost J (2006). Temporal correlation based learning in neuron models. Theory Biosci.

[CR39] Jost J (2005). Dynamical systems. Examples of complex behavior.

[CR40] Jost J (2015). Mathematical concepts.

[CR44] Jost J (2015b) Object oriented models vs. data analysis—is this the right alternative? In: Carrier M, Lenhard J (eds) Mathematics as a Tool, vol 327. Boston Studies in the Philosophy and History of Science. doi:10.1007/978-3-319-54469-4_14

[CR43] Jost J, Engel A, Friston K, Kragic D (2016). Sensorimotor contingencies and the dynamical creation of structural relations underlying percepts. The pragmatic turn. Toward action-oriented views in cognitive science.

[CR41] Jost J (2017a) Biologie und Mathematik (monograph in preparation)

[CR42] Jost J (2017b) Neural networks (monograph in preparation)

[CR45] Jost J (2017c) Relations and dependencies between morphological characters (in press) 10.1007/s12064-017-0248-z PMC5486545 28569346

[CR46] Jost J, Holthausen K, Breidbach O (1997). On the mathematical foundations of a theory of neural representation. Theory Biosci.

[CR47] Keller R (1995). Zeichentheorie.

[CR48] Kircher A (1669) Ars Magna Sciendi, sive combinatoria, Amsterdam. In: Sommer A (ed) Vienna (2005)

[CR49] Markram H, Lübke J, Frotscher M, Sakmann B (1997). Regulation of synaptic efficacy by coincidence of synaptic APs and EPSPs. Science.

[CR50] Needham (2017) Science and civilization in China, vol 2. Cambr. Univ. Press, Cambridge

[CR51] Neuser W (2013). Wissen begreifen. Zur Selbstorganisation von Erfahrung, Handlung und Begriff.

[CR52] Pfeifer R, Scheier C (1999). Understanding intelligence.

[CR53] Quine WVO (1953) From a logical point of view. Cambridge (1980)

[CR54] Rossi P (1983) Clavis universalis: Arti delle memoria e logica combinatoria da Lullo a Leibniz, Società editrice il Mulino, Bologna. English translation: Rossi P (2006) Logic and the art of memory. The quest for a universal language. Continuum, London

[CR55] Schmidt-Glintzer H (1990). Geschichte der chinesischen Literatur.

[CR56] Silver D (2016). Mastering the game of Go with deep neural networks and tree search. Nature.

[CR57] Smith JD, Minda JP (2002) Distinguishing prototype-based and exemplar-based processes in dotpattern category learning. J Exp Psychol Learn Memory Cogn 28:800–811 12109770

[CR58] Shannon C, Weaver W (1949). The mathematical theory of communication.

[CR59] Spence J (1984) The memory palace of Matteo Ricci. Penguin

[CR60] Tomasello M (1999). The cultural origins of human cognition.

[CR61] Tomasello M (2003). Constructing a language. A usage-based theory of language acquisition.

[CR62] Vapnik VN (1995) The nature of statistical learning theory. Springer, New York

[CR63] Vapnik VN (1998). Statistical learning theory.

[CR64] von Ehrenfels Chr (1980) Über “Gestaltqualitäten”, Vierteljahresschrift für wissenschaftliche Philosophie 14, pp 242—292. English translation: (1988) On ’Gestalt qualities’. In: Smith B (ed) Foundations of gestalt theory. Philosophia Verlag, München, pp 82–117

[CR65] Von Neumann J (1967) In: Burks AW (ed) Theory of self-reproducing automata. University of Illinois Press, Urbana

[CR66] Von Uexküll J, Mildenberger F, Herrmann B (2014). Umwelt und Innenwelt der Tiere. Klassische Texte der Wissenschaft.

[CR67] Weinberg S (1993) The first three minutes. A modern view of the origin of the universe, Basic Books

[CR68] Williamson T (2000). Knowledge and its limits.

[CR69] Yates F (1966). The art of memory.

[CR70] Ziemke A, Breidbach O (1996). Repräsentationismus Was sonst?.

